# No evidence of temporal preferences in caching by Western scrub-jays (*Aphelocoma californica*)^☆^

**DOI:** 10.1016/j.beproc.2013.12.010

**Published:** 2014-03

**Authors:** James M. Thom, Nicola S. Clayton

**Affiliations:** Department of Psychology, University of Cambridge, Downing Street, Cambridge CB2 3EB, UK

**Keywords:** Scrub-jay, Temporal discounting, Intertemporal choice, Self-control, Corvid, Caching

## Abstract

Humans and other animals often favour immediate gratification over long-term gain. Primates, including humans, appear more willing to wait for rewards than other animals, such as rats or pigeons. Another group displaying impressive patience are the corvids, which possess large brains and show sophisticated cognitive abilities. Here, we assess intertemporal choice in one corvid species, the Western scrub-jay (*Aphelocoma californica*). These birds cache food for future consumption and respond flexibly to future needs. Cache-theft and cache-degradation are time-dependent processes in scrub-jay ecology that might necessitate sensitivity to delays between caching and retrieval. We adopt a caching paradigm with delays of up to 49 h. Across two experiments we find no evidence of a preference for earlier recovery. We highlight the possibility that, although scrub-jays can discriminate between the present and the future, they may not understand how far into the future an event will occur.

## Highlights

• We ask whether scrub-jays prefer earlier cache-retrieval when caching.
• Caching appears similar for trays that come back after 1 h, 25 h, and 49 h.
• Caching appears similar for delays of 7 min and 26 h when no choice of trays is provided.
• We find no evidence of a preference to cache in trays that are more accessible for recovery.

## 1 Introduction

Tradeoffs between rewards at different points in time are ubiquitous in many animals’ choices about mating, cooperation, parental investment, and foraging. In an uncertain world, gains expected in the future may never bear fruit. Future gains and losses should therefore be underweighted relative to those available in the present. Indeed, many animals choose smaller, more immediately available rewards over larger, more delayed rewards, including pigeons (Ainslie, 1974; Green et al., 2004), monkeys (Addessi et al., 2011; Tobin et al., 1996), rats (Green et al., 2004; Richards et al., 1997), and humans (Green and Myerson, 2004; Kirby et al., 1999). This behaviour is commonly described in terms of ‘temporal discounting’ of future rewards; here we adopt the theoretically neutral term ‘intertemporal choice’.

Investigation of human intertemporal choice has focused primarily on monetary decisions. For example, “*Would you prefer $54 today, or $55 in 117 days?*” (taken from Kirby et al., 1999). A robust feature of human intertemporal choice is dynamic inconsistency; people are especially delay-averse as the prospect of gratification becomes more immediate. For example, many people prefer $50 immediately to $100 in six months, but would opt for $100 in a year over $50 in six months, despite the difference in delay and reward between the options being identical across the two choices. This pattern of preference is best described mathematically by a hyperbolic function (Green et al., 2004; Rachlin et al., 1991).

While humans will sometimes wait 25 years for a large (hypothetical) sum of money (Green et al., 1996), rats and pigeons are typically intolerant of delays beyond a few seconds (Green et al., 2004). Several primate species show a greater propensity to wait for a larger pay off (Addessi et al., 2011; Amici et al., 2008; Beran, 2002; Stevens and Muhlhoff, 2012; Tobin et al., 1996). Indeed, chimpanzees were *more* delay-tolerant than humans under similar task conditions in one study (Rosati et al., 2007; though see also Paglieri, 2013, for discussion of methodology).

Species differences in intertemporal choice have been attributed to a range of factors including metabolic rate (Tobin and Logue, 1994), brain size (Tobin et al., 1996), and the demands of an animal's ecology (Addessi et al., 2011; Stevens et al., 2005a,b). Corvids, like the great apes, exhibit impressive cognition across a range of physical (Bird and Emery, 2009; Taylor et al., 2007; Weir and Kacelnik, 2006) and social (Bugnyar and Heinrich, 2005; Emery and Clayton, 2001; Ostojic et al., 2013) domains, underpinned by large brains for their body sizes (Jerison, 1973). Corvids also seem to show delay tolerance comparable to that seen in great apes: carrion crows (*Corvus corone*) and common ravens (*Corvus corax*) waited for up to 320 s for a preferred reward in a delayed exchange task (Dufour et al., 2012; though see also Wascher et al., 2012 for limitations). Emery and Clayton (2004) argue that similarities in corvid and great ape psychology are the product of convergent evolution, driven by similar ecological challenges of both a physical and social nature.

Many corvids cache food for consumption at a later date. Caching inherently involves an intertemporal choice (Stevens, 2010): *eat now or cache for later*? In one species of caching corvid, the Western scrub-jay (*Aphelocoma californica*), this behaviour is mediated by separate motivational drives to eat and to cache (Clayton and Dickinson, 1999). The latter system is relatively inflexible, eliciting persistent caching in the absence of any feedback at recovery (de Kort et al., 2007). However, when given a choice of food or caching location, scrub-jays show sensitivity to the conditions of cache-retrieval, avoiding locations that have been pilfered (de Kort et al., 2007), and selectively caching food items that will be highly valued at recovery (Correia et al., 2007). This behaviour depends on input from the general satiety system governing feeding, and on prospective cognition.

Correia and colleagues manipulated subjects’ motivational states using specific satiety – the decrease in incentive value of one food type following consumption of that food type. Subjects were given the opportunity to cache, and then recover, two food types: peanuts and kibble. Pre-feeding ensured specific satiety for one food type during caching, and for the other during recovery. As found previously (Clayton and Dickinson, 1999), the birds cached fewer of the items that they were satiated on in trial 1. Importantly however, on trials 2 and 3, the birds were able to overcome their current desires and switch to caching the food they would want at recovery as opposed to that they want at the present time when caching. Another food-caching corvid, the Eurasian jay (*Garrulus glandarius*), can cache for two different future motivational states (Cheke and Clayton, 2012).

Scrub-jays are therefore able to dissociate from the context of the present and cache in accordance with a future desire to eat. This has been considered evidence of a capacity for future planning (Cheke and Clayton, 2010; Raby and Clayton, 2009), which has traditionally been thought unique to humans (Suddendorf and Corballis, 1997, 2007 but see Corballis, 2012; for full discourse see also Suddendorf and Corballis, 2008; Clayton et al., 2008). Representation of future rewards is an important mechanistic consideration for the study of intertemporal choice. At its simplest, a choice between eating and caching is governed by competition between the eating and caching drives, with no representation of the delay. This is an intertemporal choice, but not one that is sensitive to any temporal contingencies. Control of caching by predicted future drives implies representation of the *consequences* of intertemporal caching decisions. Scrub-jays are thus sensitive to delay-dependent fluctuations in the incentive values of cached food, as noted by Correia et al. (2007).

Western scrub-jays face two key ecological challenges that necessitate sensitivity to delays between caching and recovery. First, scrub-jays are versatile food-cachers. Unlike specialist cachers such as Clark's nutcrackers (Balda and Kamil, 1989), scrub-jays cache all year round and store a range of different food types (Curry et al., 2002). Degradation of dead invertebrates is considerably faster than that of nuts and seeds, and is likely to depend on seasonal variations such as humidity and temperature. Scrub-jays keep track of their caches and target recovery to items that are currently edible depending on the delay between caching and retrieval using ‘episodic-like memory’ (Clayton et al., 2001, 2003; Clayton and Dickinson, 1998; de Kort et al., 2005). They can also keep track of different foods that perish and ripen at different rates (de Kort et al., 2005). Work with another versatile cacher amongst the corvids, magpies (*Pica pica*), has also shown some aspects of episodic-like memory (Zinkivskay et al., 2009). It seems likely that sensitivity to the future incentive value of food when caching (Correia et al., 2007) would incorporate degradation and ripening. Since these are time-dependent processes, some representation of the delay between caching and retrieval would be required.

Second, scrub-jays also need to contend with cache theft, and exhibit a range of strategies to protect their stores (Dally et al., 2004, 2006a,b; Stulp et al., 2009). Some of these strategies appear highly cognitive (Emery and Clayton, 2001; Thom and Clayton, 2013). The continuous risk of pilferage should affect optimal delays between caching and retrieval (Grodzinski and Clayton, 2010): the earlier an item is recovered and eaten, the less time it has to be stolen. Scrub-jays may therefore be expected to cache with the intention of recovery in the near future. Indeed, scrub-jays typically retrieve caches more quickly than Clark's nutcrackers (Pravosudov and de Kort, 2006), despite no substantial differences in long-term spatial memory between the two species (Bednekoff et al., 1997).

In this study, we examined intertemporal choice patterns in scrub-jay caching behaviour. Experiment 1 assessed distribution of caches across three trays associated with different delays between caching and recovery. Experiment 2 tested the motivation to cache in a single tray depending on delay-to-recovery, using a shorter minimum delay than Experiment 1. Experiment 2 also introduced opportunity costs, allowing more time to recover after short delays than longer ones. In both experiments, a tendency to cache fewer items when the delay between caching and retrieval is long would indicate preferences consistent with ‘temporal discounting’ over long delays in scrub-jay caching.

## 2 Experiment 1

In Experiment 1, the birds were given the opportunity to cache mealworms across three trays, each associated with a different delay: 1 h/25 h/49 h. Mealworms are high-value food items that degrade quickly. For each mealworm, the birds had to choose (1) whether or not to cache and (2) where to cache (i.e. when to recover). We had two main predictions. First: the eating system would be delay-sensitive, and so would drive differential caching between the trays. Second: any detectable differentiation would follow a hyperbolic pattern, as seen in other species (e.g. Green et al., 2004), so the biggest difference in caching would be seen between shorter delays.

### 2.1 Materials and methods

#### 2.1.1 Ethics statement

Work was conducted under UK Home Office project licence PPL 80/2519.

#### 2.1.2 Subjects and housing

Eight hand-raised Western scrub-jays participated as subjects in this study. In both experiments, all birds had previous experience with caching and recovery (e.g. Clayton and Dickinson, 1998). Birds were fed on a maintenance diet of fruit, vegetables, mixed nuts, grains and seeds, bread, dog biscuits, and cuttlefish bone. The birds had ad libitum access to water at all times. Subjects were pair-housed in 2 m^3^ home cages, kept at 21 ± 1 °C on a 12:12 h light–dark schedule.

#### 2.1.3 Apparatus and testing conditions

Birds were given 40 mealworms to cache across three trays. One bird had previously refused to cache mealworms, and so was given 30 ‘wax worms’ (wax moth larvae) per trial instead. The worms were held in an open opaque plastic bowl in the centre of the cage floor. The three trays were placed equidistant from the bowl, against the sides of the cage.

Caching trays each consisted of an ice cube tray attached to a wooden base. Each tray was a 2 × 7 formation of potential caching sites – individual cube moulds filled with corncob caching substrate. Formations of Lego^®^ blocks around one or two sides of the wooden base were added to aid the birds’ recognition of their trays. The relationship between tray location and Lego^®^ formation was held constant for each bird across trials.

#### 2.1.4 Design

Each trial was carried out across three days. Subjects were given the opportunity to cache worms across three trays on day 1. Each bird received the same three trays on every trial. One tray was then made available for recovery on each day. Therefore, tray 1 was returned 1 h after caching, tray 2, 25 h, and tray 3, 49 h. No experimenter was present in the testing room during either caching or recovery. Caches remaining in the trays at the end of each trial were removed.

**Fig. 1:**
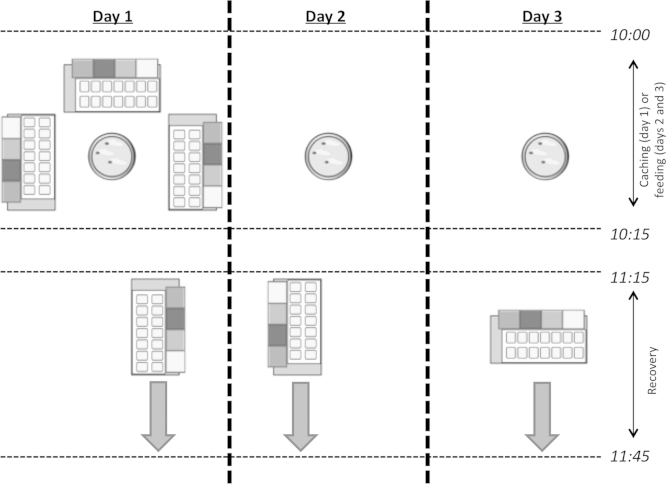
Experiment 1 trial procedure schematic. Trays are shown with Lego Duplo^®^ blocks along one edge, a 2 × 7 formation of cells, also with a bowl of worms at caching. Large arrows indicate the availability of the respective trays for recovery. Days are displayed in order from left to right. Caching (day 1) or feeding (days 2 and 3) is followed by recovery from one tray. Here, the right-hand tray is returned on day 1, the left on day 2, and the central tray on day 3.

The delays between caching and recovery for the three trays were 1 h, 25 h, and 49 h (Fig. 1). The day on which each tray (left/right/back of cage) was made available was counterbalanced between birds. The length of the recovery period was always 30 min.

Each subject completed eight trials in total. Trials were administered consecutively, with a one-day break at the end of every second trial, in accordance with UK Home Office project licence PPL 80/2519. During break days subjects did not receive either worms or caching trays, and were not isolated from their cage partner.

#### 2.1.5 Procedure

Testing began at 08:00 on day 1. Subjects were separated into adjacent visually occluded 1 m^3^ areas of the home cage. Subjects’ maintenance food was removed from the cages from 08:00 to 11:00 to motivate caching (Clayton and Dickinson, 1999).

A 15-min session of caching (on day 1) or feeding (on days 2 and 3) began at 10:00. On day 1, subjects were provided with a bowl of worms and three caching trays. On days 2 and 3, the birds were given the number of worms they were estimated to have consumed during caching on that trial (the number of worms available at the start of caching minus those found in the trays or cage after caching), and no trays. This feeding period was designed to control hunger at recovery, across days of one trial. At the end of caching/feeding, all worms and bowls were removed from the cages. Trays were then taken to a separate room where they were emptied cell by cell. The location and number of all cached worms were recorded. Trays were then refilled with substrate, and caches were returned to the cells in which they were found.

At 11:15 on all days, the birds were given 30 min to recover their stores from one tray. At 11:45, all trays were removed from the cages, maintenance food was returned, and subject pairs were reunited. Trays were checked for caches again after each recovery. Caches remaining in the trays at the end of each trial were removed.

#### 2.1.6 Analysis

A bird's preference for a tray was operationalised as the mean number of items cached in that tray across the final three trials. In many trials, no worms remained uncached and in the cage at the end of caching. This is by design: the birds had to choose when they wanted to recover a worm, rather than cache as many as possible in all trays. The number of worms cached across two of the three trays therefore provided a ceiling for caching in the third. In order to ensure all the data were sampled independently, we chose in advance to exclude all data from one tray from the headline analysis of caching. We expected the largest difference in delay to elicit the largest difference in caching, so the main comparison was between the 1-h and 49-h trays, using a planned paired samples *t*-test.

Changes in caching across the trials were assessed using post hoc single-factor (trial) repeated measures ANOVA. To avoid the independence issues outlined above, individual trays were assessed separately. Post hoc ANOVAs were carried out against a sidak-corrected type I error rate of .010. All other statistical tests in all experiments were carried against a type I error rate of .05, two-tailed.

### 2.2 Results and discussion

**Fig. 2:**
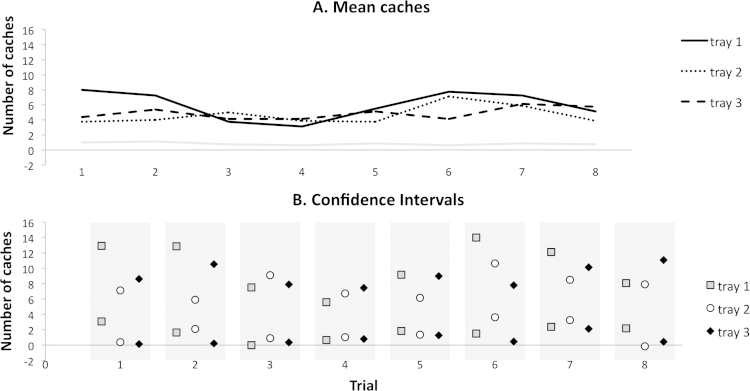
Experiment 1 caching behaviour, displayed by trial. A – mean number of caches made across birds in each tray by trial. The mean number of caches made within the cage but outside the tray is also shown. B – upper and lower bounds of 95% confidence intervals for the mean number of caches across birds, per trial.

The mean number of caches made across the final three trials did not differ significantly between the trays returned on days 1 and 3 (*t*(7) = 0.125, *p* = .904). Fig. 2 displays the mean distribution of caches on each trial; there is a high degree of overlap in the 95% confidence intervals for each tray.

If each tray is considered separately, there is no significant change in the propensity to cache in any tray across trials (tray 1 – *F*(7) = 1.015, *p* = .432; tray 2 – *F*(2.724) = 1.118, *p* = .362; tray 3 – *F*(7) = 0.257, *p* = .967). Neither was there a significant effect of trial on overall caching across the trays (*F*(3.097) = 1.361, *p* = .281). There was also no significant difference across trials if worms cached outside of the trays but in the cage were included (*F*(7) = 0.940, *p* = .485). Taken together, these results do not indicate an underlying preference between the trays, nor is there evidence of any change in overall caching.

Caching outside of the trays is consistently low throughout the experiment (Fig. 2). This is unsurprising; caching elsewhere in the cage offers unpredictable payoffs because the experimenter removed worms found cached in the cage. Furthermore, cached maintenance diet was removed at the start of each trial and could otherwise be pilfered by the bird's cage partner outside of the trial.

Overall, Experiment 1 found no evidence of a preference for a shorter delay between caching and recovery. Interpretation of a null result is difficult, but the striking overlap in confidence intervals across the trials (Fig. 2) suggests that the birds are indifferent when choosing which tray to cache in.

It seems unlikely that the birds were unable to discriminate between the trays, given previous findings (e.g. Emery and Clayton, 2001; de Kort et al., 2007; Raby et al., 2007). There are thus two plausible explanations for these data. First, the birds did not associate the trays with particular delays between caching and recovery. If so, they were either unable to discriminate between two future delays, or they had insufficient opportunity to learn the connection between delay and tray. These possibilities are raised at greater length in the general discussion.

If the birds were capable of learning when a tray would return for recovery and did so, the results of Experiment 1 might suggest no underlying preference for earlier recovery. This would be surprising given the widespread tendency for animals, including other corvids (Dufour et al., 2012), to opt for immediate receipt of rewards. However, the shortest delay used here, 1 h, is far in excess of those typically used in animal work. It may be that a preference for immediate retrieval is only detectable over shorter delays. Alternatively, caching may be a special case, impervious to the effects of delay. Experiment 2 tested these possibilities.

## 3 Experiment 2

Experiment 2 asked whether scrub-jays’ propensity to cache in a single tray varies depending on delay-to-recovery. Two delays were used: 7 min and 26 h. Experiment 2 asked two questions of the birds: (1) *Whether to cache?* and (2) *Where to cache?* (tray vs. elsewhere in the cage). Experiment 2 also introduced variable recovery session lengths. Experiment 1 offered a choice between delays until a forced recovery period, Experiment 2 varied the delay until caches *first* become available for recovery: once the tray is returned, caches remain available for recovery for the remainder of the trial. Caching when the delay is short therefore offers freer access to stores in the short-term, as would be true of caches recovered naturally. To separate preferences for longer recovery periods from those for shorter delays after caching, a third condition (“*7*-*SR*”) featured a 7-min delay but a recovery length corresponding to the 26-h condition. If the birds cache similarly in the 26-h and 7-SR conditions, then any effect of delay on caching could be attributed to a preference for longer recovery sessions, rather than for earlier recovery per se. Similar caching across conditions despite these changes would suggest that caching is truly insensitive to delay.

### 3.1 Materials and methods

#### 3.1.1 Subjects and housing

Fourteen hand-raised Western scrub-jays participated as subjects in this study, five of which had participated in Experiment 1. Three subjects were excluded from this study following repeated failure to cache; the exclusion criterion was set at failure to cache in four of five successive trials. Subjects were pair-housed in 2–4 m^3^ home cages; housing and maintenance diet was otherwise as described in Experiment 1.

#### 3.1.2 Materials

Birds were given wax worms to cache inside a tray. The worms were held in an open opaque plastic bowl, which was placed in front of a single caching tray.

#### 3.1.3 Design

**Fig. 3:**
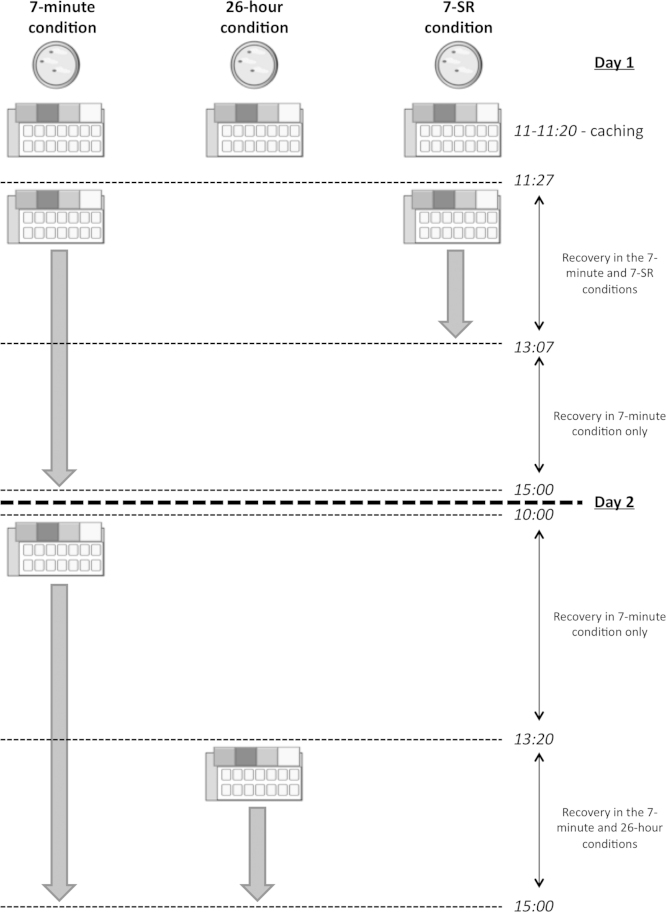
Experiment 2 trial procedure schematic. Trays are available under three recovery conditions: 7-min delay (left), 26-h delay (centre), and 7-SR (right). Large arrows indicate the availability of the tray for recovery in the respective condition.

Each trial was carried out across two days. Subjects were given the opportunity to cache wax worms in a caching tray on day 1. The rest of the trial was dedicated to cache recovery, after a specific delay. The delay between caching and recovery was either 7 min, or 26 h (see Fig. 3 for a schematic of the different delays). No experimenter was present in the testing room during either caching or recovery.

In the 7-min and 26-h conditions, once a tray was made available for recovery it remained in the cage for the rest of the trial. In 7-SR trials, the tray was made available for recovery 7 min after caching, as in the 7-min condition. However, trays in the 7-SR condition were removed 100 min after their return, as trays in the 26-h condition were.

Subjects completed 18 trials in total, blocked by delay. Delay order was counterbalanced between birds. Trials within a block were administered consecutively, with a one-day break at the end of every third trial, in accordance with UK Home Office project licence PPL 80/2519. Inter-block intervals were variable. Throughout the experiment, the tray and Lego^®^ formation received by each bird did not change in order to eliminate any potentially confounding cues.

#### 3.1.4 Procedure

Testing began at 09:00 on day 1. Subject-pairs were isolated into adjacent 1 m^3^ cages and deprived of maintenance diet.

Subjects were given 20 min to cache from 11:00 on day 1. They were provided with one caching tray and a bowl containing 20 wax worms. At the end of caching, all worms and bowls were removed from the cages, and subjects’ maintenance diet was returned.

Immediately after caching, trays were checked and re-filled; the location and number of all cached worms were recorded. Trays were returned after a specific delay from the end of caching. In the 7-min and 7-SR conditions, trays were returned at 11:27, i.e. 7 min after the end of the caching phase.

After 100 min (at 13:07), trays were removed from those birds in 7-SR trials. Testing on day 1 ended at 15:00. Trays were removed from the cages and subject pairs were reunited.

The trial recommenced at 10:00 on day 2. As on day 1, all subjects were isolated into 1 m^3^ cages. At this time, the trays were returned to those birds in the 7-min condition so they could continue recovering their caches. At 13:20 on day 2, trays were returned to subjects in the 26-h condition. At 15:00, the trial ended. All trays were removed from the cages and subject pairs were reunited. Caches remaining in the trays at the end of each trial were removed.

#### 3.1.5 Analysis

As in Experiment 1, caching across the final three trials of each block was compared by delay, using repeated measures ANOVA.

### 3.2 Results and discussion

**Fig. 4:**
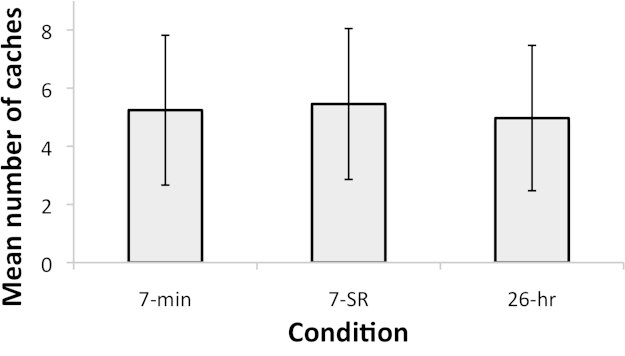
Experiment 2 results – mean number of tray caches across the final three trials of a block, by condition. Error bars indicate 95% confidence intervals.

The birds cached on average 5.24(±1.16) worms in the final three trials of the 7-min condition, 4.97(±1.12) in the 26-h condition, and 5.45(±1.17) in the 7-SR condition. In no trial did any bird cache all 20 worms. There was no significant effect of condition on level of caching (*F*(1.37, 15.07) = 0.123, *p* = .808). Substantial overlap in 95% confidence intervals (Fig. 4) is suggestive of indifference, but clearer conclusions cannot be drawn from a null result alone.

Taken together, the results of Experiment 2 provide little evidence of a preference for earlier recovery when caching. The birds did not noticeably cache more worms when the delay to recovery was 7 min than when it was 26 h. These conditions differed in two factors: the magnitude of the delay between caching and recovery and the time available to recover caches. The apparent indifference between the conditions suggests that the birds were in fact reacting to neither factor.

## 4 General discussion

Taken together, the results of this study provide no evidence for a preference for earlier recovery in Western scrub-jay caching behaviour. Overall, the birds cached for delays-to-recovery ranging from 7 min to 49 h. Experiment 1 found no difference in caching with delays of 1 h, 25 h and 49 h. The between-tray design precluded any influence of inter-trial variance, and found marked overlap in the trays’ confidence intervals across the experiment, suggesting no preference for any tray. In Experiment 2, the birds showed no evidence of increased caching in a tray with either a shorter delay-to-recovery, or a longer recovery session length.

The absence of an obvious preference for either earlier or later recovery limits the scope for influence. Scrub-jays have a natural propensity to cache, and will do so even in the absence of reinforcement from recovery (de Kort et al., 2007). Persistent and indifferent caching across delay conditions could therefore be indicative of equal preference for delays, or of no representation of delays at all.

We suggest three plausible interpretations of the results of this study. First, scrub-jays do prefer earlier recovery, but with a delay as long as 7 min (the shortest used here) the incentive value of retrieval loses its ability to motivate discriminative caching. However, this possibility is hard to square with scrub-jays’ sensitivity to future desires when caching (Correia et al., 2007). In Correia and colleagues’ study, the birds overcame their current motivational state to cache what they would want 210 min later, far longer than the shortest delay in this study: 7 min. We therefore find this explanation unlikely.

Second, the scrub-jays did not respond to the different delay conditions because they were oblivious to them. This would be true if the tasks provided insufficient prior learning experiences, or if the birds would never be likely to discriminate between future delays. Previous work demonstrates scrub-jay prospective caching on the basis of as few as three (Raby et al., 2007) or one (Correia et al., 2007) prior encounters with a tray. If the birds did not learn about the delays involved here then, it seems likely that they find learning about delays harder than learning about conditions at recovery, perhaps even impossible.

Though previous work suggests that scrub-jays can dissociate the present from the future (Correia et al., 2007), we are not aware of any evidence that they can tell *how far* into the future cache recovery will be. Recent work has shown that another corvid, the Eurasian jay, can cache for two different recovery periods associated with different desires (Cheke and Clayton, 2012; also seen in scrub-jays, Correia and Clayton, unpublished data). However, it is unclear whether the birds understood that one recovery period occurred *after* the other as opposed to just at different times with no sense of ordinality, or even whether the discrimination was primarily temporal, between recovery periods, or whether the discrimination was primarily spatial, namely between trays. If scrub-jays cannot distinguish between long future delays when caching, then they arguably cannot be said to be making intertemporal choices.

Separating a failure to learn from an inability to learn is difficult. Indeed, it is arguably impossible to demonstrate an inability to learn to discriminate future delays. We are therefore prevented from drawing conclusions beyond highlighting the relatively extensive learning experience offered in this study compared with some previous work.

The final possible explanation of our results is that scrub-jays can learn to expect a delay between caching and recovery, but are indifferent to the length of the delay. This proposal may initially seem unsurprising from a functional perspective; caching obviously has its utility in future survival, so why should it be subject to a psychological process that devalues the future recovery of caches? However, scrub-jays anticipate the context of recovery 210 min in advance, and are motivated to cache accordingly (Correia et al., 2007). If this is evidence of planning, then the relevant motivational system *must* be sensitive to expected consumption up to 210 min ahead, and can enhance caching. It is plausible that scrub-jays are limited in their capacity to foresee recovery over longer delays. Beyond their ‘temporal horizons’ (Rosati et al., 2007), the birds might rely on the simpler, inflexible caching drive alone. Selective preferences for caching when the delay to recovery is short would therefore represent the attenuated contribution of a flexible motivational system approaching and beyond the bird's temporal horizon.

The account of scrub-jay prospective caching above is speculative, but if correct then it follows that the birds’ capacity for planning is limited to outcomes in the near future. We might therefore expect more flexible responses to problems in the near future, such as the threat of cache theft by a conspecific (Emery and Clayton, 2001). However, a prospective motivational system so-limited would be unable to respond to a slow process like food-degradation. Instead, the birds would rely on retrospective episodic-like memory to drive recovery of items while they are edible.

Despite the inconclusive results of this study, we believe that caching provides a promising paradigm for the study of intertemporal choice. Caching is a naturally future-oriented behaviour, with direct consequences over long periods of time. Persistent caching in the absence of perceived reinforcement means that any reliable delay-dependent change in caching can be easily attributed to genuine intertemporal choice. This contrasts with some operant paradigms in which delay-dependent attenuation of responding might represent partial or complete degradation of instrumental knowledge. Further, previous work offers good understanding of the motivational processes incentivising caching. Incorporating the costs imposed by delayed retrieval into models of incentive values and motivation would further elucidate the mechanisms underlying caching.

In this article we have not so far referred to impulse control. Impulse control is typically important for the maintenance of intertemporal choices, and is central in some human intertemporal choice tasks (Mischel and Ebbesen, 1970; Mischel et al., 1972). Impulse control is also required in for many animal procedures, such as accumulation tasks (e.g. Beran, 2002), and the delayed exchange tasks on which corvids have previously been tested (Dufour et al., 2012). To our knowledge, there is little or no role for impulse control in decisions about where to cache. Caching is inherently future-oriented, so there is no prospect for immediate consumption in the form of eating, and no appetitive response to inhibit. However, inhibitory control may be important for cache *recovery*, since a recovered item can be eaten immediately. Like caching, recovery is an ecologically relevant intertemporal choice, and a strong drive to recover high-value food immediately would protect those stores from theft and degradation. Cache recovery may therefore represent an inviting opportunity for future work on intertemporal choice in scrub-jays. In this study however, differential recovery between the delay conditions could only affect the results if the difference was in recovery *during the caching session*. Since the caching session was always identical regardless of delay condition, we do not see a role for impulse control in the behaviour observed here.

In summary, we found no clear evidence of a preference for earlier recovery in the caching behaviour of Western scrub-jays. We suggest two likely reasons for this in light of findings from previous studies. First, the birds may have been unable to discriminate between future recovery periods on the basis of how far into those future the events would occur. Second, the birds may have learnt about the delays imposed between caching and recovery but were indifferent to them. The ability to discriminate between future rewards on the basis of delay to their receipt is essential for genuine intertemporal preferences. Future studies could clarify the results of this study by assessing the scrub-jays’ capacity to discriminate between different time points in the future. Investigation of scrub-jay intertemporal choice in a non-caching context would complement the caching studies, and would be more directly comparable with previous work using other species. Together, this work would clarify our understanding of scrub-jay intertemporal choice, and could shed some light on the adaptive function of complex cognition in caching.
